# Hospitalization Rates for Respiratory Diseases After L’Aquila Earthquake

**DOI:** 10.3390/ijerph16122109

**Published:** 2019-06-14

**Authors:** Francesco D’Aloisio, Pierpaolo Vittorini, Anna Rita Giuliani, Maria Scatigna, Jacopo Del Papa, Mario Muselli, Giorgio Baccari, Leila Fabiani

**Affiliations:** 1 Department of Life, Health and Environmental Sciences, University of L’Aquila, Piazzale Salvatore Tommasi, 1-67100 L’Aquila (AQ), Italy; pierpaolo.vittorini@univaq.it (P.V.); annarita.giuliani@univaq.it (A.R.G.); maria.scatigna@univaq.it (M.S.); leila.fabiani@univaq.it (L.F.); 2 Department of Life, Health and Environmental Sciences, Graduate School of Hygiene and Preventive Medicine, University of L’Aquila, Piazzale Salvatore Tommasi, 1-67100 L’Aquila (AQ), Italy; jacopodelpapa@hotmail.it (J.D.P.); mario.muselli@hotmail.it (M.M.); docbaccari@gmail.com (G.B.)

**Keywords:** earthquakes, respiratory diseases, hospital records

## Abstract

The study aims to investigate the impact of the earthquake on public health, in terms of hospitalizations for respiratory diseases in the Abruzzo region, focusing on the area damaged by the earthquake “Crater”. We collected data of hospitalizations of residents in Abruzzo between 2009 and 2015. Hospital Discharge Records (HDRs) with a primary diagnosis of respiratory disease were included and divided into pneumonia, Chronic Obstructive Pulmonary Disease (COPD), and respiratory insufficiency. Absolute frequencies and standardized hospitalization rates were calculated to perform both a short-term and a medium-long term analysis. A linear regression was performed using standardized hospitalization rates and the time. A total of 108.669 respiratory-related records were collected and the most frequent subgroup was respiratory insufficiency. Standardized Hospitalization Rates (SHRs) for respiratory diseases resulted higher in the non-Crater than Crater area, but the short-term analysis showed a significant increase in hospitalizations for pneumonia and respiratory insufficiency in the Crater area. The medium-long term analysis reported a significant difference on the slope decrease of hospitalizations for acute and chronic respiratory diseases in the Crater versus the non-Crater area. The earthquake may have played a triggering role in the increased detection of respiratory diseases. A temporal relationship between the quake and an increase in admissions was found although it is not yet possible to detect a direct cause-effect relationship.

## 1. Introduction

In the last decades, earthquakes have caused a large number of victims, injured, missing and displaced people, mostly in seismic areas. On 6 April 2009, the city of L’Aquila, located in central Italy, was hit by a strong earthquake that caused more than 300 deaths, mainly as the consequence of the collapse of several buildings. The effects of the earthquake on public health started to be studied both in the early phases and in terms of medium-long-term effects [1].

As of the recent literature, researchers investigated the possibility of both short-term, and long-term health effects linked to a natural disaster, such as an earthquake (e.g., [2]). A study related to the Niigata-Chuetsu earthquake compared mortality rates before and after the disaster emphasizing a significant increase in long-term mortality caused by acute myocardial infarction both in men and women [3]. Moreover, environment and pollutants exposure showed evident effects on public health. Air pollution is able to provoke acute health effects, such as including the progression of cardiovascular and respiratory diseases, acute respiratory infections and attacks of bronchial asthma, mainly caused by oxidative stress and inflammation [4]. In addition to this, several health problems were reported with the involved rescue workers in terms of mental health, such as post-traumatic stress syndrome and psychological distress [5].

Although there are strong differences among geographic areas, the effects of respiratory diseases have a significant impact on healthcare expenditures: In the world, 9 million people die from respiratory conditions each year. In Europe, respiratory conditions cause 660,000 deaths, and account for about 6 million hospital admissions, contributing to 7% of all causes of hospitalization. The current background might get worse in the near future: They are responsible for 1/10 of overall worldwide mortality, but they are expected to contribute to 1/5 by 2030 [6].

On the one hand, the fact that a certain number of patients affected by respiratory diseases were hospitalized allowed us to analyze them by means of Hospital Discharge Records (HDRs). On the other hand, the high incidence and prevalence rates represent a good base for statistical analyses and identifying potential affecting factors, like earthquakes, since they have possibly a multifactorial etiopathogenetic relationship.

HDRs, instituted in Italy with a Ministerial Decree dated 28 December 1991, are a largely diffused source of health and healthcare information. The good quality and accuracy of the information collected are obtained through a continuous and incisive monitoring system that was implemented after the agreement between the Ministry and the Regions aiming at a fruitful collaboration [7].

HDRs are maintained by hospitals primarily for management and accounting purposes, but they are currently used nowadays for epidemiological research, too [8,9].

The use of HDRs in epidemiology has strengths, limitations and may introduce biases [10]. The most important strengths are that data already exist, are large and are collected independently from the research purposes. On the other hand, the limitations are that data are pre-collected by non-researchers with a low, or unknown, quality and may lack confounders detection. 

Biases may be also present, like misclassification as the result of unclear or erroneous clinical documentation, or the fact that expensive medical procedures are usually documented better than those less costly [11]. Furthermore, other important limitations regarding HDRs are the underestimation and misclassification of the current cases, which may affect the estimation of a specific disease [12]. 

In Italy, HDRs are contained in the so-called “File A” of the health record which is divided into “File A1” (patients’ identity records) and “File A2” (patients’ health-related information) [13].

### Objectives

Our study aims to provide information about the potential influences of the earthquake of 6 April 2009 on public health, in terms of hospitalization rates in the Abruzzo region for all causes of admissions and then focus on respiratory conditions, both for acute and mid-long terms.

In detail, the objectives of our study were to compare regional hospitalizations data of the 57 municipalities damaged by the earthquake, named hereafter “Crater”, (more information in Figure S1) with the others, in terms of both a short- and a medium-long-term analysis.

For the short-term analysis, we expected an increase in acute conditions hospitalizations, such as pneumonia, around the immediate post-quake period. Most of the resident population was hosted in tents and other temporary accommodations in a still cold period of the year, because of the interruption of traditional health care services in the main hospital “San Salvatore”.

For the medium-long-term analysis, it is necessary to consider the possible influence of the deficit Repayment Plan, signed on 6 March 2007, which affected the Region. Starting in 2007, the Abruzzo region health system has faced debts and a spending review policy was required from the National government to generally reduce expenditures. For this reason, we expect an overall decrease of hospitalizations, especially for chronic conditions, which may be less pronounced, or even in countertrend, in the Crater versus non-Crater area.

## 2. Materials and Methods 

Official Ethics Committee approval was obtained from the Internal Review Board of Local Ethics Committee, L’Aquila University (ref. number 4904 dated 1 February 2018).

The study collected data regarding all kind of hospitalizations of citizen residents in the Abruzzo region in the period of time starting 1 January 2009 and ending 31 December 2015 [14]. 

HDRs were provided by the Abruzzo’s RHA (Regional Health Agency). They were collected by the Management Service of Information Flows and Health Statistics of Abruzzo Region by the file A of the HDRs.

Among the available metadata contained in File A1 and File A2, those relevant for the paper are general patient information (e.g., gender, birth date), the International Classification of Diseases (ICD9-CM) codes for the patient’s main and concurrent pathologies, and dates regarding the hospitalization and discharge.

The 9th revision of the International Classification of Diseases with Clinical Modification, (ICD-9-CM) was used to encode the diagnoses [15].

All hospital admissions of residents in Abruzzo that met the inclusion criteria for one of the primary diagnosis codes were included in the analyses. Admissions for respiratory diseases, with HDRs that reported a primary diagnosis code between 460 and 519, according to the 8th Chapter of the ICD 9-CM system, were included.

Furthermore, in our analyses, we grouped the ICD9-CM codes for respiratory disease in three main subgroups (more information available in Table S1) as follows:

- **Pneumonia**: ICD9-CM codes (480–489) were used to investigate cases in both the general population and in particular in children (0–14 years);
- **Chronic Obstructive Pulmonary Disease (COPD)**: ICD9-CM codes (490, 491, 492, 494, 496) or (786.0, 786.2, 786.4 if at the same time one of the previous codes appears as a secondary diagnosis), were used to investigate both cases in the general population, and in particular, in the elderly (aged over 65);
- **Respiratory insufficiency**: ICD9-CM codes (518.81, 518.82, 518.83, 518.84) were used to investigate both cases in the general population, and in particular, in the elderly (aged over 65).

Annual hospitalization rates were calculated and a focused analysis of the post-earthquake period was performed, investigating hospital admissions rates for respiratory disease both in a short- and a medium-long-range of time. 

The methodology of the analysis focused on both the overall number of hospitalizations and the Standardized Hospitalization Rates (SHRs), as follows:

- The overall number of hospitalizations was calculated for all causes and for the respiratory diseases, stratified by gender and age groups. As for the respiratory diseases, the frequencies were calculated both in general, by area of residence, by admission year (for the Crater area), and for the over-65 age group (for COPD and respiratory failure). The results concerning the number of hospitalizations and characteristics of the population are reported in Section 3.1 of Section Results.
- The SHRs were calculated for all causes and all respiratory conditions, as well as specifically for pneumonia, asthma, COPD, and respiratory failure for the residents of the Crater and non-Crater areas, as well as for the entire Abruzzo region. The source for population data, needed to compute the SHRs, was the Italian Institute of Statistics (ISTAT), which provided the number of residents of the region for each province, divided by municipality, gender and age group, (see Table S2). SHRs were used for both short and medium-long term analyses.

The short-term analysis consisted of:

▪ Reporting the SHRs for two months before the earthquake (i.e., February 2009 and March 2009) and six months after the earthquake (i.e., from April 2009 to September 2009);
▪ Comparing the SHRs of the Crater and non-Crater areas using confidence intervals based on the gamma distribution [16].

The medium-long-term analysis consisted of:

▪ Reporting the SHRs and their linear regressions for the years 2010–2015;
▪ Comparing the trends in the crater and non-crater areas using confidence intervals.

In both cases, the difference was considered statistically significant if the confidence intervals of Crater and non-Crater did not overlap. In the tables that report the confidence intervals, a statistically significant difference is highlighted in blue.

The results of SHRs are reported in Section 3.2 of the section Results.

The software used for the statistical analyses was R (version 3.5.3) for Linux ( Linux Foundation, San Francisco, USA).

## 3. Results

### 3.1. Total Number of Hospitalizations

Overall, 1.647.676 HDRs of Abruzzo residents were evaluated between 2009 and 2015 and about 619.000 people were hospitalized with a mild predominance of female gender: 52.9% vs. 47.1%. Their mean age was 46.96 ± 27.37 (47.54 ± 27.83 for males and 48.39 ± 26.66 for females). More details about population study data and hospital stay are shown in Table 1. HDRs caused by respiratory diseases contributed to 6.6% of all hospital admissions and 9.4% for patients older than 75 years old. Between 2009 and 2015 we evaluated 108.669 respiratory-related records and about 39.000 people were hospitalized with a mean age of 45.27 ± 32.05 (58.1% M). Respiratory insufficiency is the most frequent subgroup detected (27.1%), followed by pneumonia and COPD with 22.0% and 8.9%, respectively. More details about the total number of hospitalizations for all causes and respiratory disease by area and by year are reported in the Tables S3, S4 and S5.

Age groups analyses were performed for each respiratory disease subgroup and results showed a substantial difference among them. Respiratory insufficiency, COPD, and pneumonia involved the eldest subgroup, represented by people older than 75, in 59.9%, 53.3% and 43.0% of cases, respectively. Focusing on the year 2009, in which the disaster of the earthquake occurred, the admissions for respiratory disease were extremely higher, in people older than 75 years old (32.0%). Therefore, hospital admissions distribution for pneumonia, respiratory insufficiency, and COPD showed an evident involvement of the eldest part of the population: 38.4%, 56.3% and 56.9%, respectively, were >75 years.

### 3.2. Standardized Hospitalization Rates

#### 3.2.1. Short-Term Period

**Figure 1:**
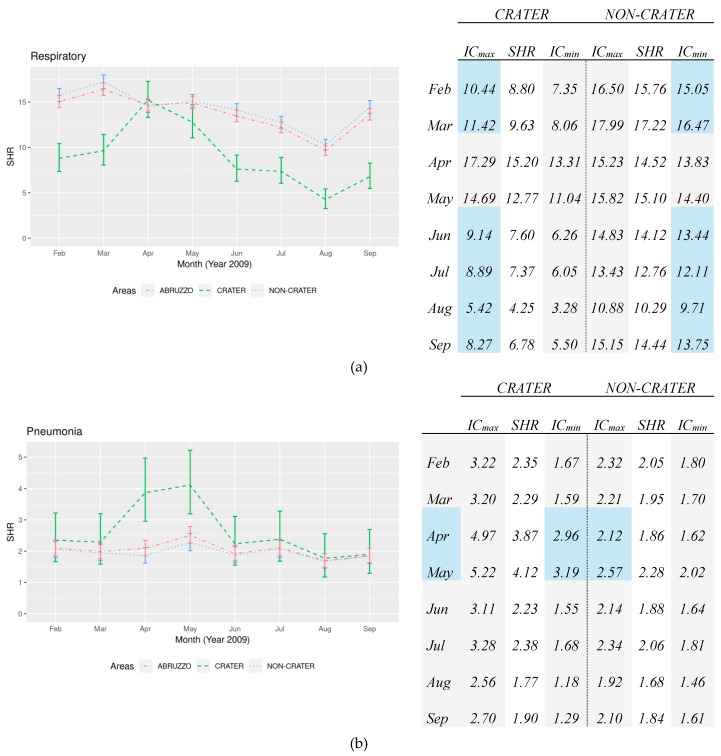

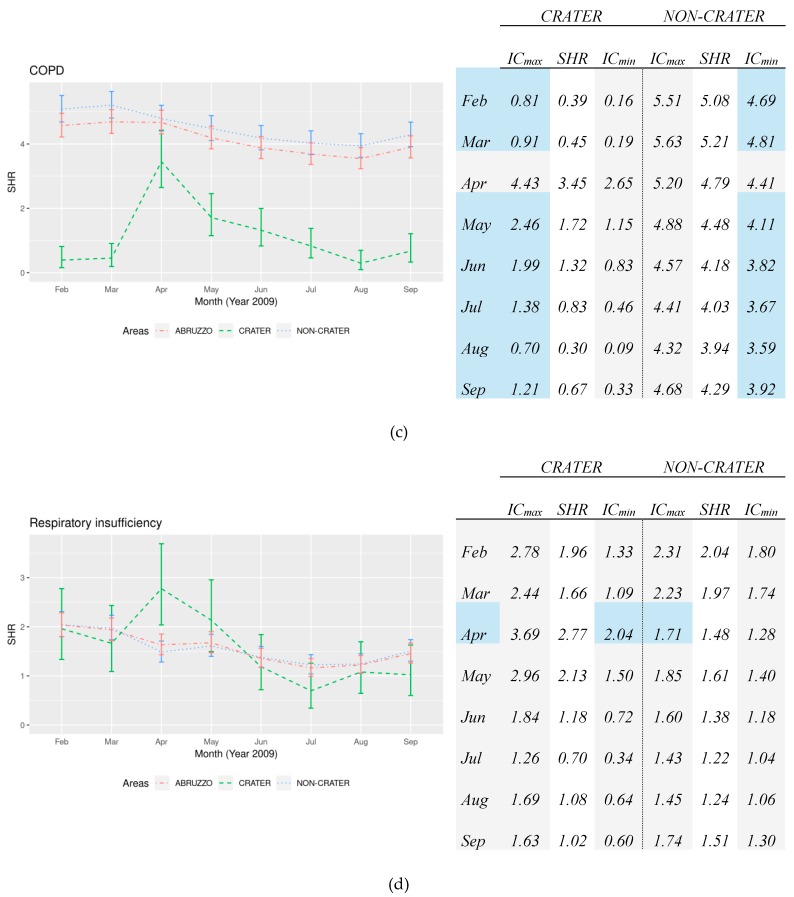
Standardized hospitalization rates (SHRs) for (**a**) respiratory disease, (**b**) pneumonia, (**c**) COPD and (**d**) respiratory insufficiency between February and September 2009 with Confidence Intervals 95%—data in the table below.

The short-term analysis of the standardized hospitalizations rates for respiratory diseases shows an overall decrease in the Abruzzo region, with substantial differences among the local areas. Respiratory diseases admissions were significantly higher in the non-Crater area vs. the Crater area, except in April and May in which we registered an overlap concerning the confidence intervals. This is due to a clearly visible increase in the Crater area in the 30-days after the earthquake of 6 April, conversely the SHRs in the non-Crater had a progressive decrease along the 8 months of analysis, as shown in Figure 1a. Pneumonia admissions rates were constantly more frequent in the Crater area respect to the non-Crater area in the short-term period (see Figure 1b). In addition, a sudden and significant increase in hospital admissions was detected in the Crater area after the quake: April monthly SHR was double that of the non-Crater one (3.87 vs. 1.86) with a significant difference. The increase persisted along the following month with a significant difference (4.12 vs. 2.28).

SHRs for COPD are significantly lower in the Crater than the non-Crater area along the short-term period analysis, except for the evident peak detected in April: (3.45 starting from 0.45 in the Crater respect to 4.79 starting from 5.21 in non-Crater) in which SHRs confidence intervals overlapped and for this the difference is not significant, as shown in Figure 1c. Moreover, the trend detected in the non-Crater was exactly the opposite respect to the one found in the Crater, a progressive decrease from March to August. Hospitalizations rates for respiratory insufficiency were constantly higher in the non-Crater area vs. the Crater area even if the difference was not significant, except in April and May. SHRs resulted significantly higher in the Crater area respect to the non-Crater area in April: 2.77 vs. 1.48. This trend persisted also in May (Crater 2.13 vs. non-Crater 1.61), although the difference reported was not significant, as shown in Figure 1d.

#### 3.2.2. Medium- and Long-Term Period

The background of the standardized hospitalizations rates for all causes of hospital admissions shows an overall decrease in the Abruzzo Region, with significant differences among the local areas. In terms of medium-long term analysis, the trend decrease for all causes of hospitalization appears less evident in the Crater area respect to the non-Crater area. The slope decrease showed a significant difference between Crater (−0.011/month) and non-Crater (−0.024/month), as shown in Table 2.

**Figure 2:**
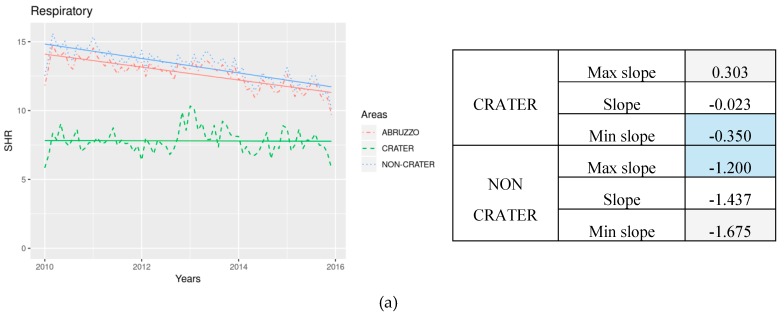

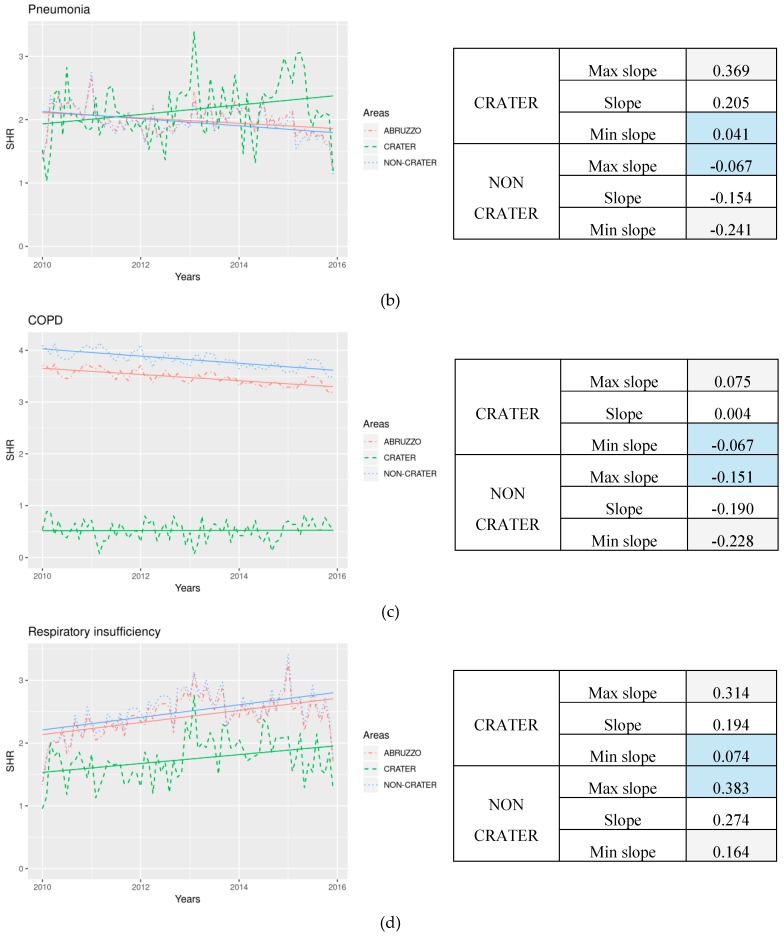
Medium-long term analysis of SHRs regression line, coefficients (slope x 1000) for (**a**) respiratory diseases, (**b**) pneumonia, (**c**) COPD and (**d**) respiratory insufficiency, between 2010 and 2015 with Confidence Intervals—data in the adjacent table.

This pattern was even more noticeable in the medium-long term for respiratory diseases (−1.286 × 10^3^/month in Abruzzo and −0.023 × 10^3^/month in Crater area), with a significant difference between the slope coefficients between Crater (−0.023 × 10^3^) and non-Crater (−1.437 × 10^3^), as shown in Figure 2a. Also, regarding pneumonia admissions, a slight decrease in the medium-long period was found in the Abruzzo Region (−0.116 × 10^3^/month), conversely with respect to the pattern found in the Crater area (+0.205 × 10^3^/month). A significant difference was found in the slope decrease: Pneumonia admissions had a positive slope in the Crater area (+0.205 × 10^3^/month) with respect to the decreasing trend in non-Crater (−0.154 × 10^3^/month), Figure 2b. The same difference and opposite trends were found with COPD admissions. Although the regional trend was on the decrease (−0.164 × 10^3^/month) in the long-term analysis, it was found to be on the increase in the Crater vs. non-Crater areas with a statistically significant difference in slope coefficients (CR 0.004 × 10^3^/month vs. NC −0.190 × 10^3^/month), as shown in Figure 2c. Conversely, hospital admissions for respiratory insufficiency reported a slight overall increase in the Abruzzo region of 0.264 × 10^3^/month. The crater area had a lower increase with respect to the non-Crater area, even if the difference found in the slope increase was not significant (CR 0.194 × 10^3^/month vs. NC 0.274 × 10^3^/month), as shown in Figure 2d.

## 4. Discussion

As shown above, the results emerging from our findings on the analyses of the HDRs highlight the following aspects. In terms of SHRs, the results that showed relevant differences are:

- Admissions for respiratory diseases involved mainly the frailest part of the population, as >75 years for respiratory insufficiency and COPD;
- SHRs resulted higher in the non-Crater than the Crater area both for all causes, for respiratory diseases, COPD, except for pneumonia which was found higher in the Crater area, and for respiratory insufficiency that resulted borderline;
- The short-term period after the quake showed a visible increase of Crater admissions for respiratory diseases within all subgroups. Monthly SHRs resulted significantly higher in the Crater than the non-Crater area for pneumonia both in April and May, and for respiratory insufficiency only in April;
- The medium-long term analysis reported an overall decrease of hospitalizations in the Abruzzo region, both for all causes, respiratory diseases, pneumonia, and COPD. Only respiratory insufficiency admissions increased. This trend is confirmed out of the area hit by the earthquake (non-Crater), while it is significantly different by the pattern found in the Crater area;
- The crater area showed a significantly less marked decrease with respect to the non-Crater area for all kind of hospitalizations, for respiratory diseases, COPD, and pneumonia. Overall, the slope decrease in the Crater area was always lower than the non-Crater area, even opposite in the case of pneumonia and COPD.

The differences detected in SHRs trends, by area, may have been caused by different factors (the earthquake at the end of the section), as discussed below.

### 4.1. Health Policy

In the years following 2007, the Abruzzo region health system fell into debt and a spending review policy was demanded by the National government to generally reduce costs due to health care. It required a strong reorganization of the hospital network, with the conversion of some of the main ones, re-qualification of home care, containment of the pharmaceutical expenditure, and the reduction of the number of beds in the hospitals. Such a spending review plan could have been the main reason for the decreasing trend of hospitalizations in the Abruzzo Region, but the differences found between the different areas might have been caused also by different factors, such as:

• Different Implementation of Community Care

For many respiratory diseases, the implementation of community care is a valuable method for reducing hospitalizations and the SHR [17]. Therefore, a different trend in the different areas could be caused by a stronger or weaker implementation of such practices. In our case, since the Crater and non-Crater areas do not belong to the same Local Health Units, community care could have had unidentical organizations and applications, with different outcomes. Therefore, such a different implementation might be a concause of the different trends in the two areas.

• Inappropriate Hospitalizations

Hospitalizations for COPD are identified at high risk of inappropriateness and therefore they are normally discouraged. This situation is very likely to be one of the causes of the decreasing trend for COPD in the Abruzzo region, indicating the decrease in inappropriate hospitalizations and perhaps a shift toward respiratory insufficiency admissions. Brainstorming with doctors working in the pneumology wards of the hospitals belonging to the internal areas of Abruzzo (larger and including Crater) highlighted the existence of strict guidelines finalized to reduce inappropriate hospitalizations due to COPD in favor of community care. This may explain both the generally lower values of SHRs for COPD in the Crater area (Figure 1c and Figure 2c), and also that during the month of April 2009 such a difference was not present, before returning to almost the same pre-earthquakes values, due to the temporary unavailability of home care.

### 4.2. Earthquake, April 2009

The increase of the number of hospitalizations for respiratory disease due to environmental disasters is documented by several studies. A review conducted by Kalantar Motamedi et al. on the earthquakes occurred in the past decade (Iran, China, Haiti, Pakistan, Peru), showed that the respiratory infectious diseases of both the upper and lower tract, especially bronchitis and pneumonia, were the most frequent conditions [18]. In Pakistan, about 3.6% of hospitalizations in the first three weeks after the earthquake were caused by lower respiratory tract infections or asthma [19]. Ochi et al. confirmed that the healthcare system is expected to answer an overload for respiratory hospitalizations in 10–30 days after the natural disaster [20]. Ohkouchi et al. found the respiratory hospitalizations registered the following month after the earthquake in Japan (Sendai, 2011) 2.7 times higher with respect to the same period of the previous year [21]. Examining the same earthquake, Yamanda et al. identified pneumonia, acute exacerbation of COPD, and asthma, as the most frequent causes of hospitalizations for respiratory disease in the two months following the event [22]. These findings are confirmed by the review performed by Murakami et al. on the Japan earthquake. They reported that respiratory disease articles were the most frequent found in their review together with mental health ones [23]. Recently, a study conducted by Fabiani et al. used HDRs for reporting a preliminary analysis to investigate the respiratory health data in terms of hospitalizations for pneumonia, asthma, COPD, and respiratory insufficiency [24]. On one hand, we partially confirmed what Ochi and Japanese researchers reported regarding the short-term effects, but on the other hand, differently from Japanese studies, we were focused on a longer span of time. Kawano et al. reviewed the medical charts of evacuees and reported acute respiratory infections as the most frequent disease at shelters (168.8 per week per 1000 evacuees) after the earthquake of Japan, 2011 [25].

According to our findings, people living in the Crater area, exposed to the quake, reported an increased SHR in the 30-days period after the event. It could have caused, in agreement with Ochi and other studies [20], a significant increase of hospital admissions expected with a peak of exacerbations of chronic conditions, like COPD, after a 10–30 days period. We found a similar situation for an acute condition, such as pneumonia and respiratory insufficiency. Our findings are confirmed along this line with a longer effect on the hospitalization trend than COPD. Our results reported actually that the highest value was registered in the month of May, rather than April that was characterized by the peak after the event.

Another issue to be taken into account is the air quality and the massive exposure to air pollution, smoke, and dust over a long period as consequence of the collapse of many buildings. Brackbill et al. reported that children and adolescents were more susceptible to a worsening of respiratory symptoms after the collapse of the World Trade Center in New York on 11 September 2001 [26]. Several studies have focused on the possible relationship between the COPD exacerbation and the exposure to air pollution, such as Belleudi who reported an increase in hospitalizations for heart failure and COPD in Rome in association with ultrafine concentration [27,28].

Comorbidities of chronic conditions should be considered as well. We included in our study hospitalizations that mention a respiratory disease as primary diagnosis. This inclusion criterion caused the loss of cases in which respiratory diseases were annotated in HDRs as a complication or an underlying medical condition in one of the five secondary diagnoses. These cases may be frequent, as mentioned by Baldo et al., who reported a comorbidity in 46.4% of patients hospitalized for pneumonia [29]. Several studies reported that a stressful event, like a quake, could play a trigger role and re-activate silent or well-controlled conditions. Cardiovascular and cerebrovascular diseases, diabetes, and hypertension may affect the risk of mortality in people with chronic respiratory diseases or worsen the asymptomatic state well-managed by the current therapy [30,31,32,33].

A further and more detailed study detecting the potential effect of concurrent diagnoses, or that include them, may be desirable and worthy of attention.

## 5. Conclusions

Although the respiratory diseases group is considered a multifactorial condition, our findings suggest that the earthquake in 2009 may have played a role in increased detection. Exacerbations of silent or sub-clinic chronic conditions, such as COPD, could be facilitated by factors able to unmask morbidity and a natural event like the earthquake may have had a triggering role in hospital admissions increases.

Based on our findings, a temporal relationship between the exposure to the natural disaster and the evidence of an increase in admissions, in a short-term and a medium-long term regarding respiratory chronic and acute diseases, was found. Furthermore, the less marked decrease in the Crater area with respect to the non-Crater area could be considered in this perspective, since both areas are under the same reduction and repayment plan health policy. 

Our study is in line with the findings of other studies [20,24] and a temporal connection between the event and the surplus of hospitalization was found. Nevertheless, it is desirable that researchers’ efforts focus on more detailed analyses, such as the trend of respiratory diseases in rescue workers, and further studies detecting several aspects, such as pollution exposure, temporary accommodations where people were forced to stay, and health facility conditions.

## Figures and Tables

**Table 1:** Mean age of the population hospitalized by cause of admission.

Population Study	Abruzzo (Mean ± SD)	Crater (Mean ± SD)	Non-Crater (Mean ± SD)	*p*-Value
All causes (*n* = 619,011)	46.96 ± 27.37	49.03 ± 26.69	46.71 ± 27.44	<0.001
Males 45.6% (*n* = 324,243)	46.31 ± 27.89	48.56 ± 27.24	46.05 ± 27.95	
Females 54.4% (*n* = 387,710)	47.41 ± 26.92	49.39 ± 26.23	47.17 ± 26.99	
M/F *p*-Value	<0.001	<0.001	<0.001	
Respiratory diseases (*n* = 38,916)	45.27 ± 32.05	52.44 ± 32.04	44.53 ± 31.95	<0.001
Males 56.9% (*n* = 26,387)	44.16 ± 30.93	51.23 ± 31.07	43.43 ± 30.82	
Females 43.1% (*n* = 19,965)	46.83 ± 33.41	54.04 ± 33.22	46.08 ± 33.34	
M/F *p*-Value	<0.001	0.009	<0.001	

**Table 2:** Medium-long term analysis of SHRs regression line, coefficients (slope x 1000), for all causes between 2010 and 2015 with Confidence Intervals—data in the table below.

Area	Slope Coefficient (CI 95%)	
CRATER	Max slope	−0.005
	Slope	−0.011
	Min slope	−0.018
NON CRATER	Max slope	−0.019
	Slope	−0.024
	Min slope	−0.029

## Supplementary Materials

The following are available online at https://www.mdpi.com/1660-4601/16/12/2109/s1, Figure S1: List of municipalities belonging to the Crater area, Table S1: ICD9-CM codes of respiratory disease subgroups, Table S2: Residents data in Abruzzo region in 2009, stratified by area, Table S3: Total number of hospitalizations for all causes by area and by year in Abruzzo region, Table S4: Total number of hospitalizations for respiratory diseases between 2009–2015, by area, Table S5: Total number of hospitalizations for respiratory diseases between 2009–2015 in Abruzzo, by year.
