# Supplementary material for: Hospitalization Rates for Respiratory Diseases After L’Aquila Earthquake
Source: Int J Environ Res Public Health. 2019 Jun 14;16(12):2109. doi: 10.3390/ijerph16122109 (PMC6616506; doi:10.3390/ijerph16122109)
Supplement: Supplementary file 1 [file ijerph-16-02109-s001.pdf]

SUPPLEMENT 1

Table S1: ICD9- CM codes of respiratory disease subgroups

ICD9-CM PRIMARY DIAGNOSES CODES	
	**CODES**
RESPIRATORY DISEASES	460-519, 786.0, 786.2, 786.4
DISEASE SUBGROUP	
*COPD**	490, 491, 492, 494, 496 or 786.0, 786.2, 786.4 (if at the same time one of the previous 490, 491, 492, 494, 496 codes appears as secondary diagnosis)
PNEUMONIA	480 - 489
RESPIRATORY INSUFFICIENCY	518.81, 518.82, 518.83, 518.84

***COPD= Chronic Obstructive Pulmonary Disease**

Table S2: Residents data in Abruzzo region in 2009, stratified by area

AGE GROUP	CRATER	NON-CRATER	ABRUZZO
0-4	5604	51723	57327
5-9	5663	51790	57453
10-14	5943	53777	59720
15-19	6743	60501	67244
20-24	7848	65516	73364
25-34	19800	158039	177839
35-44	21686	188839	210525
45-54	21156	166292	187448
55-64	17908	142474	160382
65-74	15094	121739	136833
75-84	12242	95187	107429
≥85	5006	34105	39111
TOTAL	144693	1189982	1334675

*Non-Crater is the whole Abruzzo region except the 57 municipalities recognized as “Crater”, as shown below.

Figure S1: List of municipalities belonging to the Crater area

Province of L'Aquila	Province of Teramo	Province of Pescara
1. Acciano<br>2. Barete<br>3. Barisciano<br>4. Bugnara<br>5. Cagnano Amiterno<br>6. Campotosto<br>7. Capestrano<br>8. Capitignano<br>9. Caporciano<br>10. Carapelle Calvisio<br>11. Castel del Monte<br>12. Castel di Ieri<br>13. Castelvecchio Calvisio<br>14. Castelvecchio Subequo<br>15. Cocullo<br>16. Collarmele<br>17. Fagnano Alto<br>18. Fontecchio<br>19. Fossa<br>20. Gagliano Aterno<br>21. Goriano Sicoli<br>22. L'Aquila<br>23. Lucoli<br>24. Montereale<br>25. Navelli<br>26. Ocre<br>27. Ofena<br>28. Ovindoli<br>29. Pizzoli<br>30. Poggio Picenze<br>31. Prata d'Ansidonia<br>32. Rocca di Cambio<br>33. Rocca di Mezzo<br>34. San Demetrio ne' Vestini<br>35. San Pio delle Camere<br>36. Sant'Eusanio Forconese<br>37. Santo Stefano di Sessanio<br>38. Scoppito<br>39. Tione degli Abruzzi<br>40. Tornimparte<br>41. Villa Sant'Angelo<br>42. Villa Santa Lucia degli Abruzzi	1. Arsita<br>2. Castelli<br>3. Colledara<br>4. Fano Adriano<br>5. Montorio al Vomano<br>6. Penna Sant'Andrea<br>7. Pietracamela<br>8. Tossicia	1. Brittoli<br>2. Bussi sul Tirino<br>3. Civitella Casanova<br>4. Cugnoli<br>5. Montebello di Bertona<br>6. Popoli<br>7. Torre de' Passeri

Table S3: Total number of hospitalizations for all causes by area and by year in Abruzzo region

YEAR	CRATER	NON CRATER	ABRUZZO
2009	26643	240645	267288
2010	26369	232880	259249
2011	26943	227550	254493
2012	25846	210902	236748
2013	27222	221435	248657
2014	21034	166828	187862
2015	22419	170960	193379
TOTAL	176476	1471200	1647676

Table S4: Total number of hospitalizations for respiratory diseases between 2009-2015, by area

**Group**	**Crater**	**Non-Crater**	**Abruzzo**
Pneumonia	3065	20867	23932
COPD	891	8813	9704
Respiratory insufficiency	2463	27026	29489
Respiratory diseases	10420	98249	108669

Table S5: Total number of hospitalizations for respiratory diseases between 2009-2015 in Abruzzo, by year

**Group**	**2009**	**2010**	**2011**	**2012**	**2013**	**2014**	**2015**	**Total**
Pneumonia	3698	3506	3431	3199	3559	3470	3069	23932
COPD	2580	1459	1407	1148	1150	981	979	9704
Respiratory insufficiency	2895	3532	4056	4547	4854	4808	4797	29489
Respiratory diseases	17346	16371	16160	15424	15863	13957	13548	108669
